# Anti‐growth and pro‐apoptotic effects of dasatinib on human oral cancer cells through multi‐targeted mechanisms

**DOI:** 10.1111/jcmm.16782

**Published:** 2021-07-28

**Authors:** Nam‐Sook Park, Yu‐Kyung Park, Anil Kumar Yadav, Young‐Min Shin, David Bishop‐Bailey, Jong‐Soon Choi, Jong Wook Park, Byeong‐Churl Jang

**Affiliations:** ^1^ Department of Molecular Medicine College of Medicine Keimyung University Daegu Korea; ^2^ Department of Dentistry College of Medicine Keimyung University Daegu Korea; ^3^ North Cornwall Research Institute Cornwall UK; ^4^ Biological Disaster Analysis Group Division of Convergence Biotechnology Korea Basic Science Institute Daejeon Korea; ^5^ Graduate School of Analytical Science and Technology Chungnam National University Daejeon Korea; ^6^ Department of Immunology College of Medicine Keimyung University Daegu Korea

**Keywords:** apoptosis, dasatinib, HIF‐1α, HSC‐3, Src, YD‐38

## Abstract

Dasatinib is an inhibitor of Src that has anti‐tumour effects on many haematological and solid cancers. However, the anti‐tumour effects of dasatinib on human oral cancers remain unclear. In this study, we investigated the effects of dasatinib on different types of human oral cancer cells: the non‐tumorigenic YD‐8 and YD‐38 and the tumorigenic YD‐10B and HSC‐3 cells. Strikingly, dasatinib at 10 µM strongly suppressed the growth and induced apoptosis of YD‐38 cells and inhibited the phosphorylation of Src, EGFR, STAT‐3, STAT‐5, PKB and ERK‐1/2. In contrast, knockdown of Src blocked the phosphorylation of EGFR, STAT‐5, PKB and ERK‐1/2, but not STAT‐3, in YD‐38 cells. Dasatinib induced activation of the intrinsic caspase pathway, which was inhibited by z‐VAD‐fmk, a pan‐caspase inhibitor. Dasatinib also decreased Mcl‐1 expression and S6 phosphorylation while increased GRP78 expression and eIF‐2α phosphorylation in YD‐38 cells. In addition, to its direct effects on YD‐38 cells, dasatinib also exhibited anti‐angiogenic properties. Dasatinib‐treated YD‐38 or HUVEC showed reduced HIF‐1α expression and stability. Dasatinib alone or conditioned media from dasatinib‐treated YD‐38 cells inhibited HUVEC tube formation on Matrigel without affecting HUVEC viability. Importantly, dasatinib's anti‐growth, anti‐angiogenic and pro‐apoptotic effects were additionally seen in tumorigenic HSC‐3 cells. Together, these results demonstrate that dasatinib has strong anti‐growth, anti‐angiogenic and pro‐apoptotic effects on human oral cancer cells, which are mediated through the regulation of multiple targets, including Src, EGFR, STAT‐3, STAT‐5, PKB, ERK‐1/2, S6, eIF‐2α, GRP78, caspase‐9/3, Mcl‐1 and HIF‐1α.

## 1 INTRODUCTION

Oral squamous cell carcinoma (OSCC) accounts for 24% of all head and neck cancers.^1^ OSCC is caused predominantly through exposure to areca quid chewing, cigarette smoking and alcohol consumption.^2^, ^3^ OSCC not only causes significant mortality but is associated with functional alterations in the oromaxillo‐facial region and disfigurement.^4^ The main treatments for OSCC are surgery, radiotherapy alone, or in combination with chemotherapy.^5^, ^6^, ^7^ However, despite extensive research on treatments for oral cancers, overall survival for patients with OSCC has not improved.^8^ Therefore, there is a need for both new therapies and biomarkers for OSCC progression.

Src is a non‐receptor protein tyrosine kinase,^9^, ^10^ whose overexpression and hyperactivity are associated with tumour mass, metastasis, recurrence, angiogenesis and survival of patients with cancer.^14^, ^15^, ^16^ Of further note, studies have previously demonstrated that Src is overexpressed in OSCC, including tongue cancer, and there is a significant association of Src overexpression with progression, recurrence and prognosis of OSCC.^11^, ^17^, ^18^ These findings strongly suggest Src inhibition as a targeted therapy for solid cancers, including tongue cancer.

Dasatinib (BMS‐354825) is an inhibitor of Bcr‐Abl and Src kinases that has been shown to be an effective treatment of chronic myeloid leukaemia (CML) and Philadelphia chromosome–positive acute lymphoblastic leukaemia.^19^, ^20^ Moreover, dasatinib has anti‐proliferative, anti‐invasive, anti‐angiogenic and pro‐apoptotic properties in solid tumours.^21^, ^22^, ^23^ A wealth of information strongly supports that dasatinib is a multi‐targeted tyrosine kinase inhibitor. Dasatinib inhibits the activity of Bcr‐Abl and Src family kinases along with additional receptor/non‐receptor tyrosine kinases and signalling protein kinases, including c‐KIT, platelet‐derived growth factor receptor (PDGFR), epidermal growth factor receptor (EGFR), the ephrin‐A receptor, TEC family kinases and ERK‐1/2.^24^, ^25^, ^26^ These results strongly indicate that dasatinib's anti‐tumour effects on haematological malignancies and solid cancers are largely associated with its multi‐targeted tyrosine kinase inhibition.

At present, the anti‐cancer effect and mechanism of action of dasatinib in human OSCC are not known. In this study, we investigated the inhibitory effects of dasatinib on the growth of four different human oral cancer cell lines (YD‐8, YD‐10B, YD‐38 and HSC‐3) that originated from the tongue (YD‐8, YD‐10B, HSC‐3) or lower gingiva (YD‐38).^27^, ^28^ YD‐8 and YD‐38 cells are non‐tumorigenic, whereas YD‐10B and HSC‐3 cells are highly tumorigenic.^28^, ^29^ Here, we demonstrate that dasatinib has strong anti‐growth, anti‐angiogenic and pro‐apoptotic effects on YD‐38 and HSC‐3 cells, and these effects are mediated through modulation of the expression and phosphorylation of multiple targets, including Src, EGFR, STAT‐3, STAT‐5, PKB, ERK‐1/2, S6, eIF‐2α, GRP78, caspase‐9/3, Mcl‐1 and HIF‐1α.

## 2 MATERIAL AND METHODS

### 2.1 Chemicals and antibodies

Dasatinib was purchased from LC Laboratories. Roswell Park Memorial Institute (RPMI)‐1640 medium, Dulbecco's modified Eagle's medium (DMEM), foetal bovine serum (FBS), penicillin‐streptomycin and phosphate‐buffered saline (PBS) were bought from WelGENE. 3‐(4,5‐dimethylthiazol‐2‐y)‐5‐(3‐carboxymethoxyphenyl)‐2‐(4‐sulfophenyl)‐2H‐tetrazolium (MTS) reagent was from Promega. Bradford reagent was from Bio‐Rad. Protease inhibitor cocktail (PIC, 100×) and z‐VAD‐fmk (627610) were purchased from Calbiochem. Control siRNA (sc‐37007) and Src (sc‐29228) were obtained from Santa Cruz Biotechnology. Enzyme‐linked chemiluminescence (ECL) Western detection reagents were bought from Thermo Scientific. PCR primers were purchased from Bioneer. LY294002 and PD98059 were from Biomol Research Lab. Cell culture plastic wares were purchased from SPL Life Sciences. A detailed list of antibodies used in this study is included in Supplementary Table S1.

### 2.2 Cell culture

Different OSCC (YD‐8, YD‐38 and YD‐10B) cells were procured from the Korean Cell Line Bank. HSC‐3, another human OSCC cell, was bought from American Type Culture Collection. These cells were grown in RPMI‐1640 (YD‐8, YD‐38, and YD‐10B) or DMEM (HSC‐3) supplemented with 10% heat‐inactivated FBS, 100 U/mL penicillin and 100 µg/mL streptomycin at 37ºC in a humidified atmosphere with 5% CO_2_.

### 2.3 Cell viability and survival assay

For cell viability assay, cells (1 × 10^4^ cells/mL/100 μL/well) were seeded in a 96‐well plate. After overnight incubation, cells were treated with dasatinib (0, 0.1, 1, and 10 µM) for 24 hours. Cells were then washed twice with PBS, and viability was determined using MTS reagent according to the manufacturer's protocol. Briefly, eight µl of the culture media (RMPI‐1640 or DMEM) and 20 µL of MTS solution was added to each well, and the plate was incubated at 37℃ for 1 hour. The absorbance of each well was measured at 490 nm using a microplate reader (SPECTRA max 340PC; Molecular Devices, LLC). For cell survival, cells (2 × 10^5^ cells/ml/500 μL/well) were seeded in a 24‐well plate overnight. Cells were treated with dasatinib (0, 0.1, 1, and 10 µM) for 24 hours. Cells were then washed twice with PBS. The number of survived cells, which cannot be stained with trypan blue dye, was counted using a phase‐contrast microscope. The cell count assay was performed in triplicate. Data are mean ± standard error (SE) of three independent experiments. Survival is expressed as a percentage of control.

### 2.4 DNA fragmentation assay

DNA fragmentation was performed as mentioned in our previous study.^30^ Genomic DNA was extracted and analysed via electrophoresis at 100 V on a 1.7% agarose gel for 20 minutes. The DNA was visualized and photographed under UV illumination after staining with ethidium bromide (0.1 µg/mL) using a gel documentation system (Gel Doc‐XR, Bio‐Rad Laboratories, Inc.).

### 2.5 Western blot analysis

After treatments, cells were lysed in a modified radioimmunoprecipitation assay buffer (Sigma‐Aldrich; Merck) containing PIC (1×). Proteins (50 µg) were separated by sodium dodecyl sulphate‐polyacrylamide gel electrophoresis and transferred onto nitrocellulose membranes (Millipore). The membranes were blocked with 5% (w/v) skim milk in Tris‐buffered saline (TBS) containing 0.1% Tween 20 (TBST) for 2 hours. The membranes were incubated with specific antibodies listed in Supplementary Table 1 at 4 ºC overnight. The membranes were then washed twice with TBST and exposed to secondary antibodies conjugated to horseradish peroxidase (HRP) for 2 hours at room temperature, and immunoreactivity was detected by ECL reagents. β‐actin was used as an equal protein loading control.

### 2.6 Reverse transcription‐polymerase chain reaction (RT‐PCR)

After treatments, total cellular RNA was isolated using TRIzol reagent (Life Technologies) according to the manufacturer's protocol. Complementary DNA was then prepared using M‐MLV reverse transcriptase (Gibco‐BRL) according to the manufacturer's protocol. The primer sequences used in this study are listed in Table S2. The PCR conditions applied herein were as follows: Mcl‐1, 25 cycles of denaturation at 95℃ for 45 seconds, annealing at 56℃ for 45 seconds and extension at 72℃ for 45 seconds; β‐actin, HIF‐1α and HIF‐1β, 25 cycles of denaturation at 95℃ for 30 seconds, annealing at 56℃ for 30 seconds and extension at 72℃ for 30 seconds. The amplified products were separated by electrophoresis on a 1.2% agarose gel and detected using a gel documentation system (Gel Doc‐XR, Bio‐Rad Laboratories, Inc.).

### 2.7 siRNA transfection

Small interfering RNA (siRNA) of Src (sc‐29228) and control siRNA (sc‐37007) were obtained from Santa Cruz Biotechnology. YD‐38 cells were transfected with 100 pM of control or Src siRNA using Lipofectamine RNAiMAX (Invitrogen) for 48 hours. The cells were then lysed, and protein expressions were analysed by Western blotting.

### 2.8 Measurement of HIF‐1α protein stability

YD‐38 cells were initially grown for 8 hours in culture media (without dasatinib) to induce high expression levels of HIF‐1α protein. YD‐38 cells were then treated without or with dasatinib (10 μM), PP1 (25 μM), LY294002 (25 μM) or PD98059 (50 μM) for 5, 10, 15 and 30 minutes in the absence or presence of cycloheximide (CHX), a translation inhibitor. At each time point, whole‐cell lysates were prepared and analysed by Western blotting to measure the amounts of HIF‐1α and HIF‐1β proteins remained in the cells.

### 2.9 Measurement of HUVEC tube formation

YD‐38 cells were treated without or with dasatinib or phorbol 12‐myristate 13‐acetate (PMA) at the indicated doses for 24 hours. The conditioned media (CM) was then collected and applied to human umbilical vein endothelial cells (HUVEC), which were kindly provided by professor Ho‐Jung Kwon at the Department of Biotechnology in Yonsei University, cultured in Matrigel‐coated plates for an additional 16 hours. Changes in cell morphology (HUVEC tube formation) were captured using an inverted microscope (Nikon Eclipse TS200; Nikon Corp.).

### 2.10 Statistical analyses

Results are expressed as mean ± standard error (SE) of three independent experiments. The significance of the difference between sample means was determined by one‐way ANOVA, followed by Dunnett's post hoc test, using SPSS 11.5 software (SPSS, Inc.). The difference was considered statistically significant for the value of *P* < .05.

## 3 RESULTS

### 3.1 Dasatinib inhibits growth and induces apoptosis of YD‐38 human oral cancer cells

**FIGURE 1:**
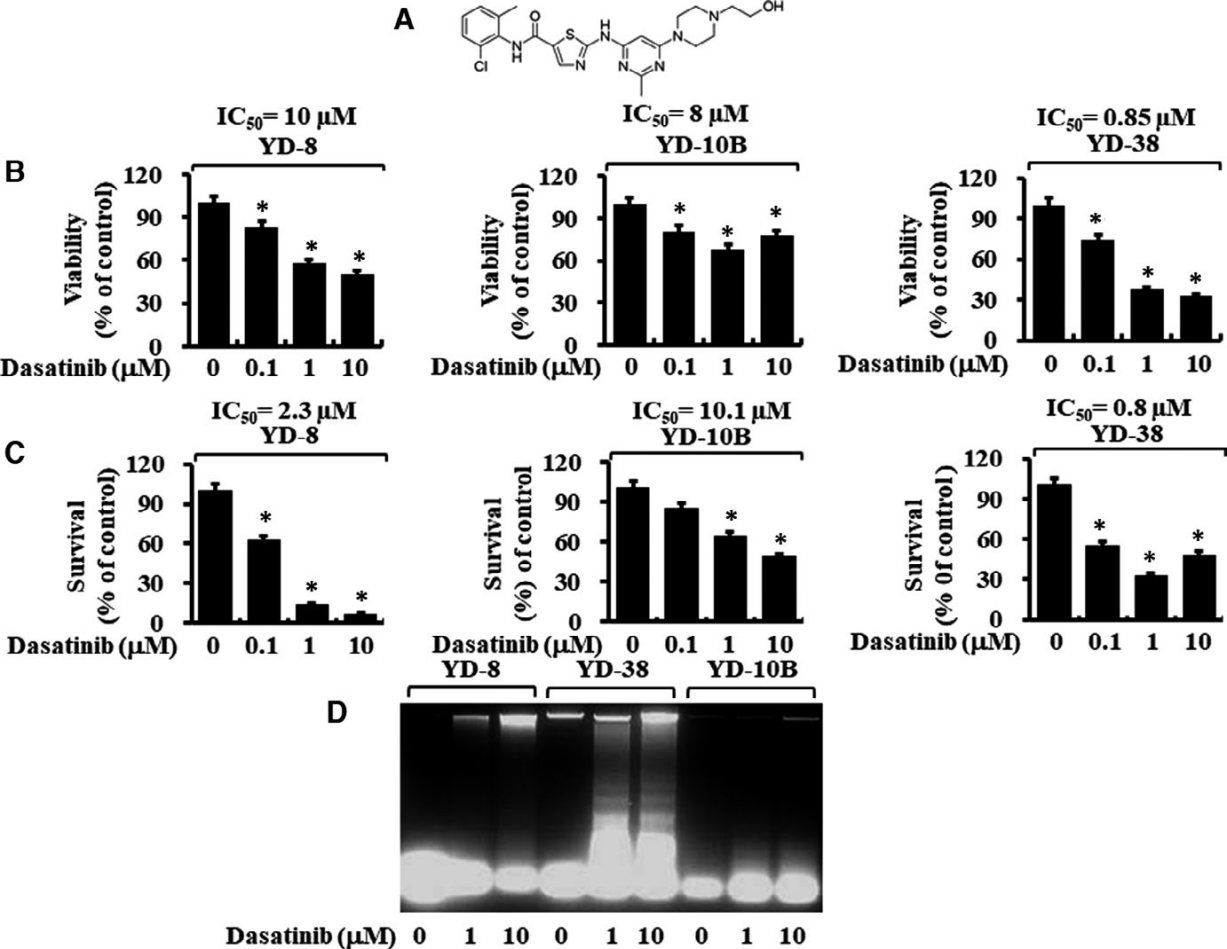
Effects of dasatinib on cell viability, cell survival and apoptosis (DNA fragmentation) on three different human oral cancer cells. A, The chemical structure of dasatinib. B‐C, YD‐8, YD‐38 and YD‐10B cells were treated with dasatinib (0, 0.1, 1 and 10 μM) for 24 h, followed by measurement of cell viability by MTS assay (B) and cell survival by cell count analysis (C). Data are mean ±SE of three independent experiments. *P < .05 compared with the values of control (no dasatinib). D, YD‐8, YD‐38 and YD‐10B cells were treated with dasatinib (0, 1 and 10 μM) for 8 h. Extra‐nuclear fragmented DNA was extracted and analysed on a 1.7% agarose gel

We initially investigated the effect of 0.1, 1 or 10 µM of dasatinib (Figure 1A) for 24 hours on the viability, survival and apoptosis of YD‐8, YD‐10B and YD‐38 cell lines. Dasatinib induced a concentration‐dependent decrease in the viability of all cell lines tested by MTS assay, with YD‐38 cells exhibiting the highest degree of sensitivity followed by YD‐10B and YD‐8 cells (Figure 1B). By contrast, using trypan blue dye–based cell count analysis, YD‐8 cells were the most sensitive to dasatinib, followed by YD‐38 and YD‐10B cells (Figure 1C). Strikingly, dasatinib only induced a concentration‐dependent accumulation of fragmented DNA in YD‐38 cells (Figure 1D). There was little or no DNA fragmentation in YD‐8 or YD‐10B cells treated with 10 µM of dasatinib for up to 8 hours.

### 3.2 Dasatinib treatment at 10 µM inhibits the phosphorylation of Src, EGFR, STAT‐3, STAT‐5, PKB and ERK‐1/2 in YD‐38 human oral cancer cells

**FIGURE 2:**
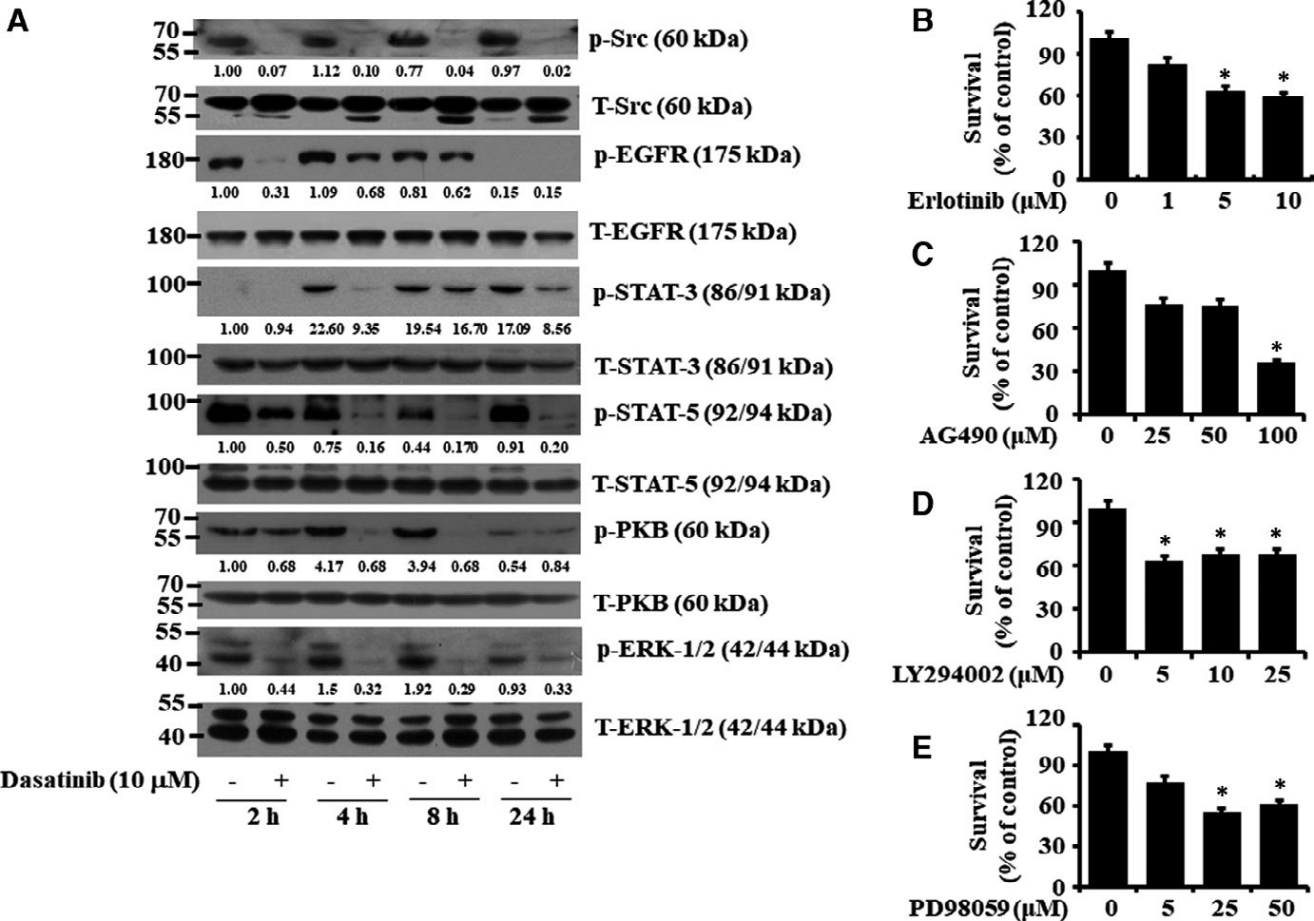
Effects of dasatinib, erlotinib, AG490, LY294002 or PD98059 on expression and phosphorylation of Src, EGFR, STAT‐3, STAT‐5, PKB and ERK‐1/2 in YD‐38 cells and cell survival. A, YD‐38 cells were treated with dasatinib (10 μM) for the indicated times. At each time point, whole‐cell lysates were prepared and analysed by Western blotting. Each picture is representative of three independent experiments. B‐E, YD‐38 cells were treated for 24 h with the indicated doses of erlotinib (B), AG490 (C), LY294002 (D) and PD98059 (E), followed by measurement of cell survival by cell count analysis. Data are mean ±SE of three independent experiments. *P < .05 compared with the values of control (no drug)

Given that dasatinib is a multi‐targeted protein kinase inhibitor, we next investigated the treatment effects of dasatinib at 10 µM on the expression and phosphorylation levels of its target Src and its related receptor kinases, signalling kinases and transcription. There were high phosphorylation levels of Src, EGFR, PKB, ERK‐1/2, STAT‐3 and STAT‐5 in YD‐38 cells in the absence of dasatinib at all time points tested (Figure 2A). Dasatinib treatment by 2 hours led to almost complete loss of the phosphorylated Src in YD‐38 cells, supporting the drug's efficacy. Furthermore, dasatinib treatment (10 µM) strongly reduced levels of the phosphorylated EGFR, STAT‐3, STAT‐5, PKB and ERK‐1/2 in YD‐38 cells at all time points tested. Expression levels of total Src, EGFR, STAT‐3, STAT‐5, PKB and ERK‐1/2 remained unchanged under these experimental conditions. We next sought to explore the role of EGFR, STAT‐3/5, PKB and ERK‐1/2 in the growth of YD‐38 cells using each of the selective pharmacological inhibitors: erlotinib (an EGFR inhibitor), AG490 (a JAK/STAT inhibitor), LY294002 (a PI3K/PKB inhibitor) and PD98059 (a MEK 2 and ERK‐1/2 inhibitor). As shown in Figure 2B‐E, individual treatments of erlotinib, AG490, LY294002 and PD98059 all led to a substantial decrease in YD‐38 cell survival.

### 3.3 Knockdown of Src causes a strong inhibition of the phosphorylation of EGFR, STAT‐5, PKB and ERK‐1/2 in YD‐38 human oral cancer cells

**FIGURE 3:**
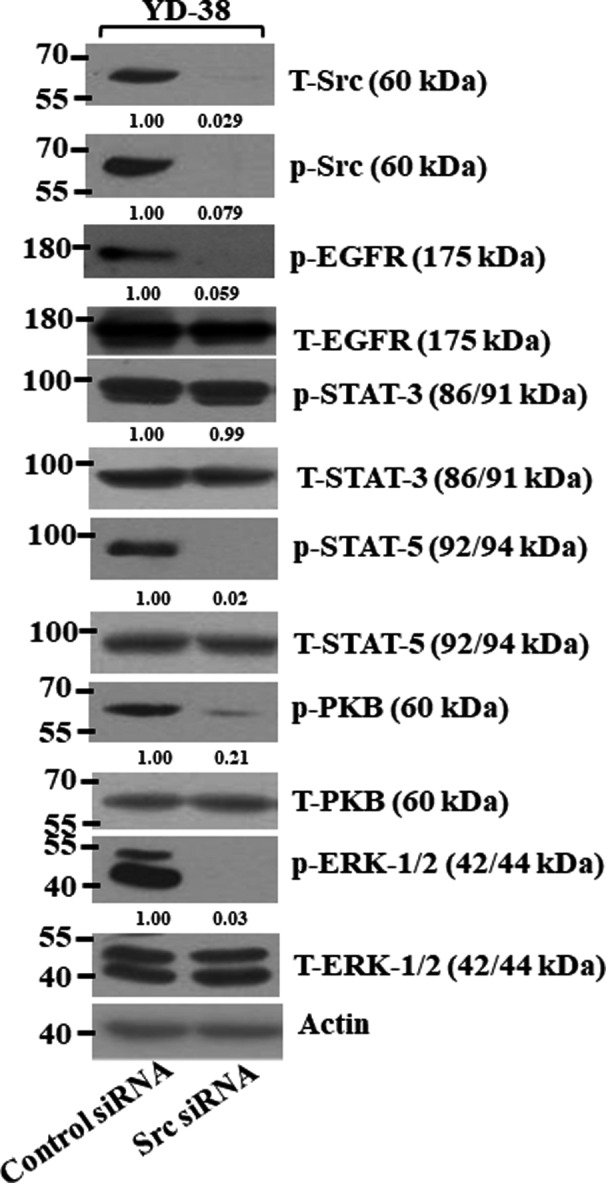
Effects of Src knockdown on expression and phosphorylation of Src, EGFR, STAT‐3, STAT‐5, PKB and ERK‐1/2 in YD‐38 cells. YD‐38 cells were transfected with 100 pM of control or Src siRNA for 48 h. Whole‐cell lysates were prepared and analysed by Western blotting. Each picture is representative of three independent experiments

We hypothesized that dasatinib‐mediated decline of the phosphorylation levels of EGFR, STAT‐3, STAT‐5, PKB and ERK‐1/2 in YD‐38 cells might be mediated through its inhibition of Src. To test this, we transfected YD‐38 cells with control or Src siRNA for 48 hours and analysed the expression and phosphorylation levels of EGFR, STAT‐3, STAT‐5, PKB and ERK‐1/2 in control or Src siRNA–transfected cells by Western blotting. High expression and phosphorylation levels of Src, EGFR, STAT‐3, STAT‐5, PKB and ERK‐1/2 were observed in control siRNA‐transfected YD‐38 cells (Figure 3). By contrast, in YD‐38 cells transfected with Src siRNA, there was little or no expression of total or phosphorylated, supporting a high Src siRNA knockdown efficiency. Strikingly, compared with control siRNA–transfected cells, there was also a marked reduction of the phosphorylation levels of EGFR, PKB, ERK‐1/2 and STAT‐5, but not STAT‐3, in these Src siRNA–transfected YD‐38 cells. Total expression levels of EGFR, STAT‐3, STAT‐5, PKB and ERK‐1/2 remained unchanged under these experimental conditions.

### 3.4 Dasatinib induces apoptosis via activation of the intrinsic caspase pathway in YD‐38 human oral cancer cells

**FIGURE 4:**
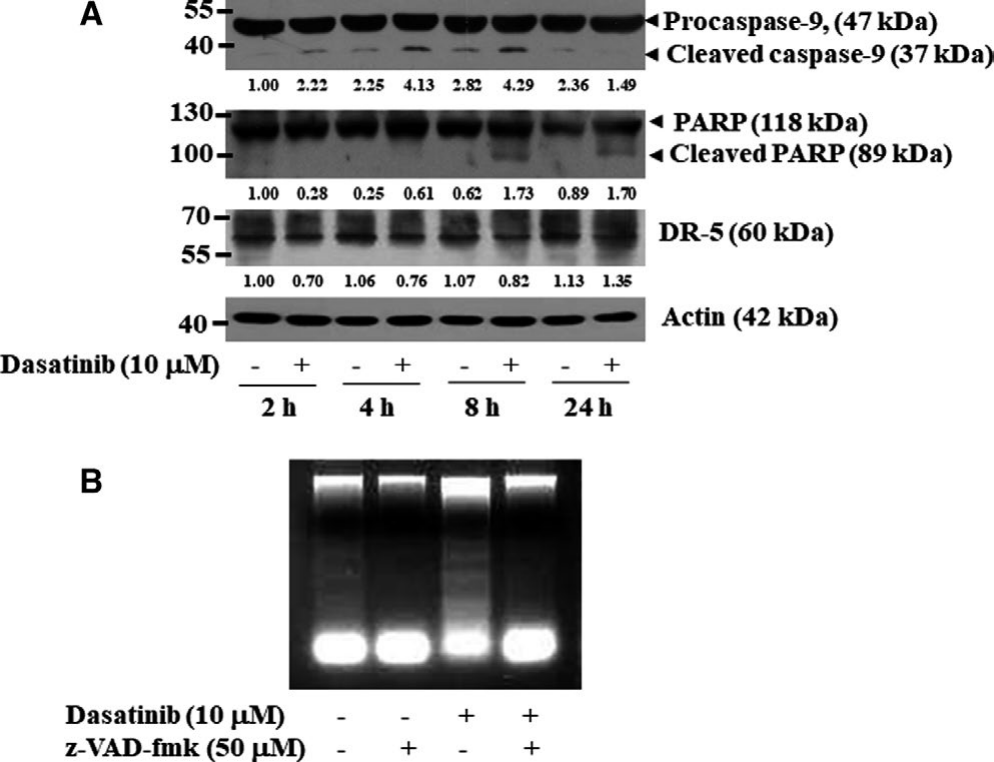
Effects of dasatinib and/or z‐VAD‐fmk on the expression of procaspase‐9, DR‐5 and PARP and DNA fragmentation in YD‐38 cells. A, YD‐38 cells were treated without or with dasatinib (10 μM) for the indicated times. At each time point, whole‐cell lysates were prepared and analysed by Western blotting. B, YD‐38 cells were pre‐treated for 1 h with a pan‐caspase inhibitor z‐VAD‐fmk (50 μM) and then treated with or without dasatinib (10 μM) in the absence or presence of z‐VAD‐fmk for an additional 8 h. The genomic DNA was extracted and analysed on 1.7% agarose gels for DNA fragmentation. Each picture is representative of three independent experiments

Activation of caspases is a principal feature for the execution of cell death by various apoptotic stimuli. Dasatinib treatment led to a time‐dependent increase in cleavage of caspase‐9 and its target PARP in YD‐38 cells (Figure 4A). To see whether dasatinib treatment modulates the extrinsic apoptotic pathway, we next measured the expression levels of death receptor‐5 (DR‐5). Dasatinib treatment did not increase the expression levels of DR‐5 in the cells; rather, a slightly decreased protein expression was observed. In further support of dasatinib actions via the intrinsic pathway, z‐VAD‐fmk, a pan‐caspase inhibitor, greatly attenuated the DNA fragmentation induced by dasatinib in YD‐38 cells (Figure 4B).

### 3.5 Dasatinib alters expression and phosphorylation levels of Mcl‐2, GRP78, eIF‐2α and S6 in YD‐38 human oral cancer cells

**FIGURE 5:**
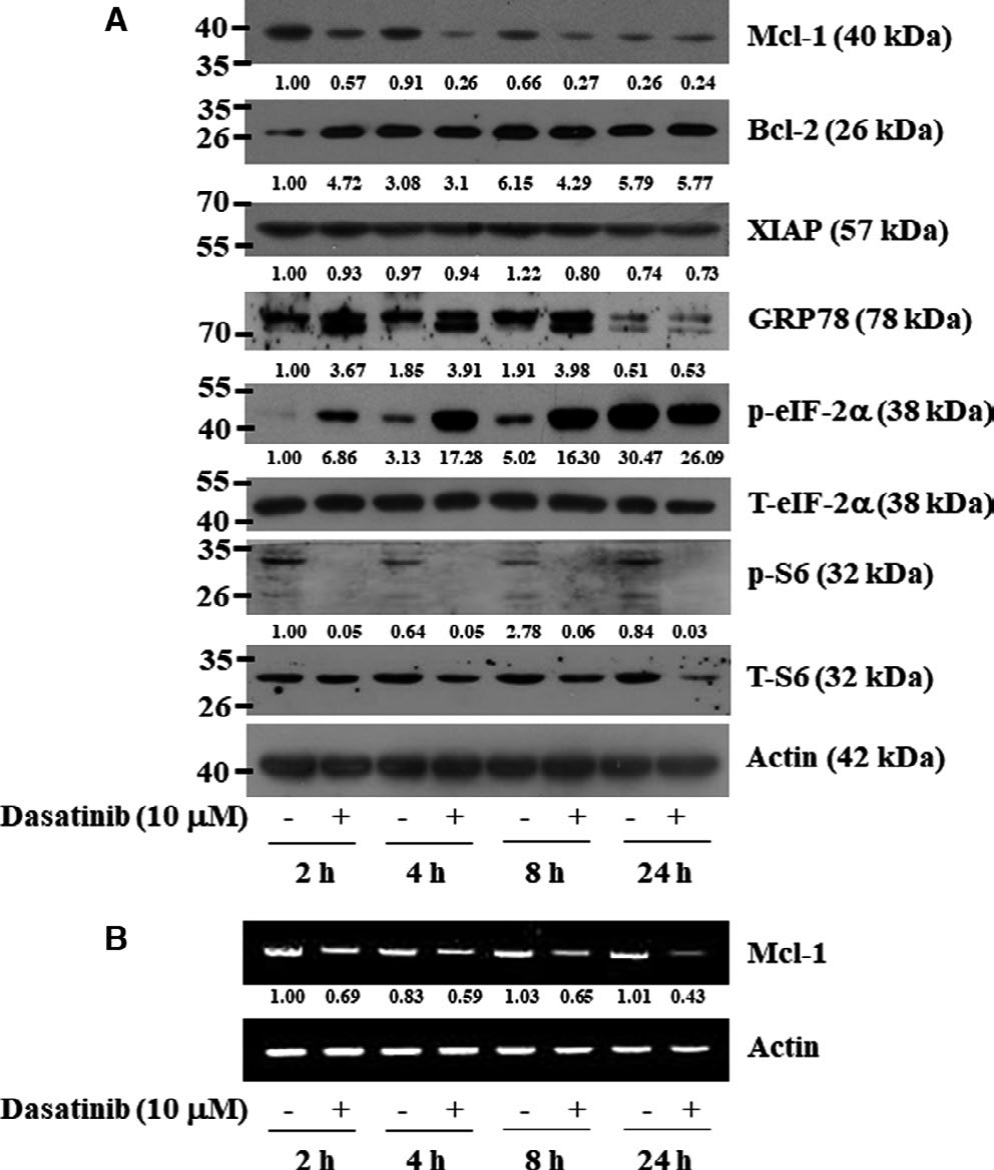
Effects of dasatinib on expression and phosphorylation of Mcl‐1, Bcl‐2, XIAP, GRP78, eIF‐2α and S6 in YD‐38 cells. A, YD‐38 cells were treated with dasatinib (10 μM) for the indicated times. At each time point, whole‐cell lysates were prepared and analysed by Western blotting. Each picture is representative of three independent experiments. B, YD‐38 cells were treated with dasatinib (10 μM) for the indicated times. At each time point, total cellular RNA was prepared and analysed by RT‐PCR analysis. Each picture is representative of three independent experiments

Given that cancer cell growth or apoptosis is influenced by the expression levels of anti‐apoptotic proteins, such as the family of Bcl‐2 and IAPs,^31^, ^32^ we next investigated whether dasatinib affects the expression levels of Bcl‐2, Mcl‐1 and XIAP in YD‐38 cells. High expression levels of Bcl‐2, Mcl‐1 and XIAP proteins were observed in untreated YD‐38 cells (Figure 5A). Dasatinib treatment led to a time‐dependent reduction of Mcl‐1 expression but did not influence the expression of Bcl‐2 and XIAP in YD‐38 cells for the times applied. RT‐PCR analysis further revealed that dasatinib reduced mRNA expression levels of Mcl‐1 (Figure 5B), suggesting Mcl‐1 transcriptional down‐regulation by dasatinib. ER stress also mediated cancer cell growth or death.^33^ GRP78 and eIF‐2α are known as ER stress markers.^34^ There was a large increase in levels of GRP78 in YD‐38 cells treated with dasatinib for 2, 4 and 8 hours. Furthermore, a time‐dependent increase in the phosphorylation levels of eIF‐2α occurred in YD‐38 cells treated with dasatinib. A ribosomal protein S6 is a component of the 40S ribosomal small subunit and is involved in translation.^35^ Notably, as further shown in Figure 5A, while dasatinib treatment for 2 hours led to reduced phosphorylation but not total levels of S6, the drug treatment for 4 to 24 hours resulted in reduced phosphorylation and total levels of S6 in YD‐38 cells.

### 3.6 Dasatinib reduces HIF‐1α at the protein levels in YD‐38 human oral cancer cells

**FIGURE 6:**
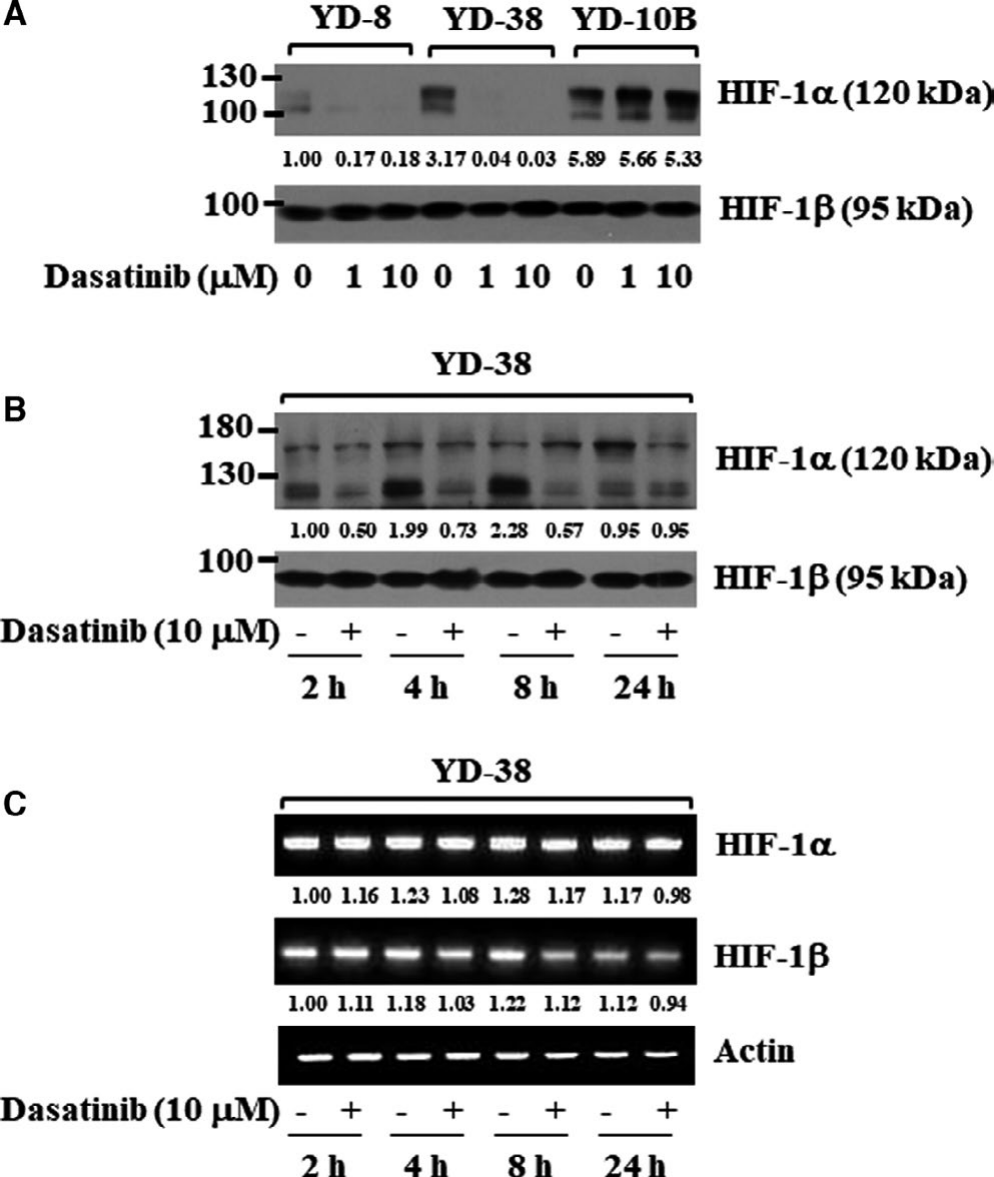
Effects of dasatinib on the expression of HIF‐1α in YD‐8, YD‐10B and YD‐38 cells. A, YD‐8, YD‐38 or YD‐10B cells were treated with dasatinib at the indicated concentrations for 8 h. Whole‐cell lysates were prepared and analysed by Western blotting. B‐C, YD‐38 cells were treated with dasatinib (10 μM) for the indicated times. At each time point, whole‐cell lysates and total cellular RNA were prepared and analysed by Western blotting (B) and RT‐PCR analysis (C), respectively

HIF‐1α is an angiogenic transcription factor.^36^ The HIF‐1α expression is linked to the tumour promotion in human oral cancer^37^ and correlates with the growth and adhesion in human OSCC cells.^38^, ^39^ In this study, we further determined whether HIF‐1α protein is expressed in YD‐38 cells and whether dasatinib modulates it. Of interest, HIF‐1α was highly expressed in YD‐38 and YD‐10B cells, but there was a low expression of HIF‐1α in YD‐8 cells (Figure 6A). Notably, dasatinib treatment at 1 or 10 µM resulted in almost complete down‐regulation of HIF‐1α protein in YD‐38 and YD‐8 cells but had no effect on the expression of HIF‐1α protein in YD‐10B cells. Furthermore, dasatinib treatment at 10 μM greatly down‐regulated HIF‐1α at the protein, but not mRNA, levels in YD‐38 cells at times tested, suggesting post‐transcriptional regulation of HIF‐1α by dasatinib (Figure 6B,C).

### 3.7 Dasatinib induces Src‐, PI3K/PKB‐ and ERK‐1/2‐dependent HIF‐1α destabilization in YD‐38 human oral cancer cells

**FIGURE 7:**
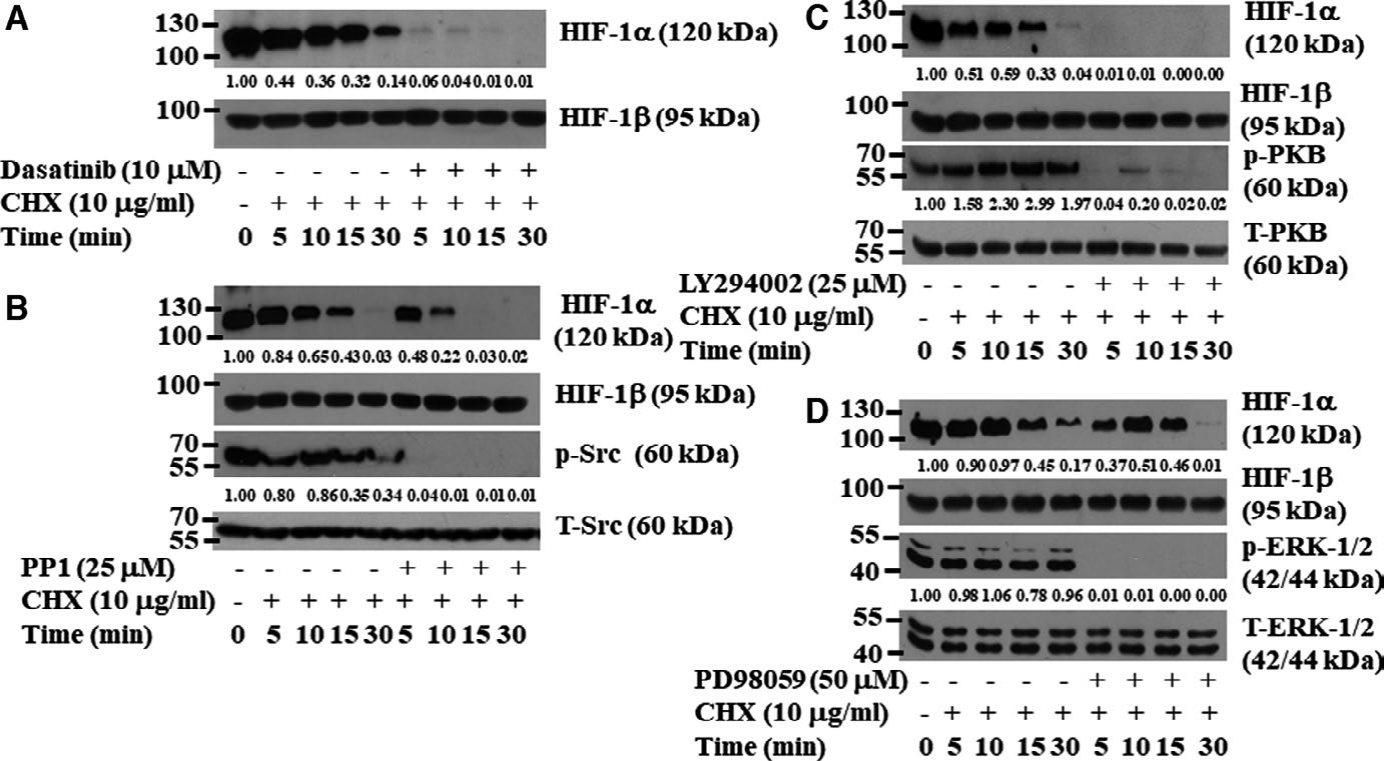
Effects of dasatinib, PP1, LY29402, or PD98059 on HIF‐1α protein stability in YD‐38 cells. A, YD‐38 cells were initially grown for 8 h in culture media to induce high HIF‐1α protein. YD‐38 cells were then treated without or with dasatinib (10 μM) for 5, 10, 15, and 30 min in the absence or presence of CHX, a translation inhibitor. At each time point, whole‐cell lysates were prepared and analysed by Western blotting. (B‐D) YD‐38 cells were initially grown for 8 h in culture media to induce high levels of HIF‐1α protein. YD‐38 cells were then treated without or with PP1 (25 μM) (B), LY294002 (25 μM) (C), or PD98059 (50 μM) (D) for 5, 10, 15, and 30 min in the absence or presence of CHX. At each time point, whole‐cell lysates were prepared and analysed by Western blotting

We next determined whether dasatinib affects the stability of HIF‐1α protein in YD‐38 cells using a CHX‐based protein stability assay. There was a gradual time‐dependent decrease in the amounts of HIF‐1α protein in YD‐38 cells grown in the presence of CHX at times tested (as shown in Figure 7A), suggesting that if the translation is blocked, HIF‐1α protein is intrinsically unstable in these cells. Dasatinib treatment caused a rapid destabilization (or degradation) of HIF‐1α protein in YD‐38 cells at all the time points tested. Given that Src, PI3K/PKB and ERK‐1/2 regulate HIF‐1α protein stability (stabilization),^40^, ^41^ and dasatinib inhibits Src, PKB and ERK‐1/2 in YD‐38 cells, we investigated the effects of PP1 (a Src inhibitor), LY294002 (a PI3K/PKB inhibitor) and PD98059 (an ERK‐1/2 inhibitor) on HIF‐1α protein stability in YD‐38 cells. Treatment with PP1 (Figure 7B), LY294002 (Figure 7C) and PD98059 (Figure 7D) decreased HIF‐1α protein in YD‐38 cells. Expression levels of control HIF‐1β and total Src, PKB and ERK‐1/2 proteins remained constant under these experimental conditions.

### 3.8 Dasatinib blocks tube formation of human umbilical vein endothelial cells

**FIGURE 8:**
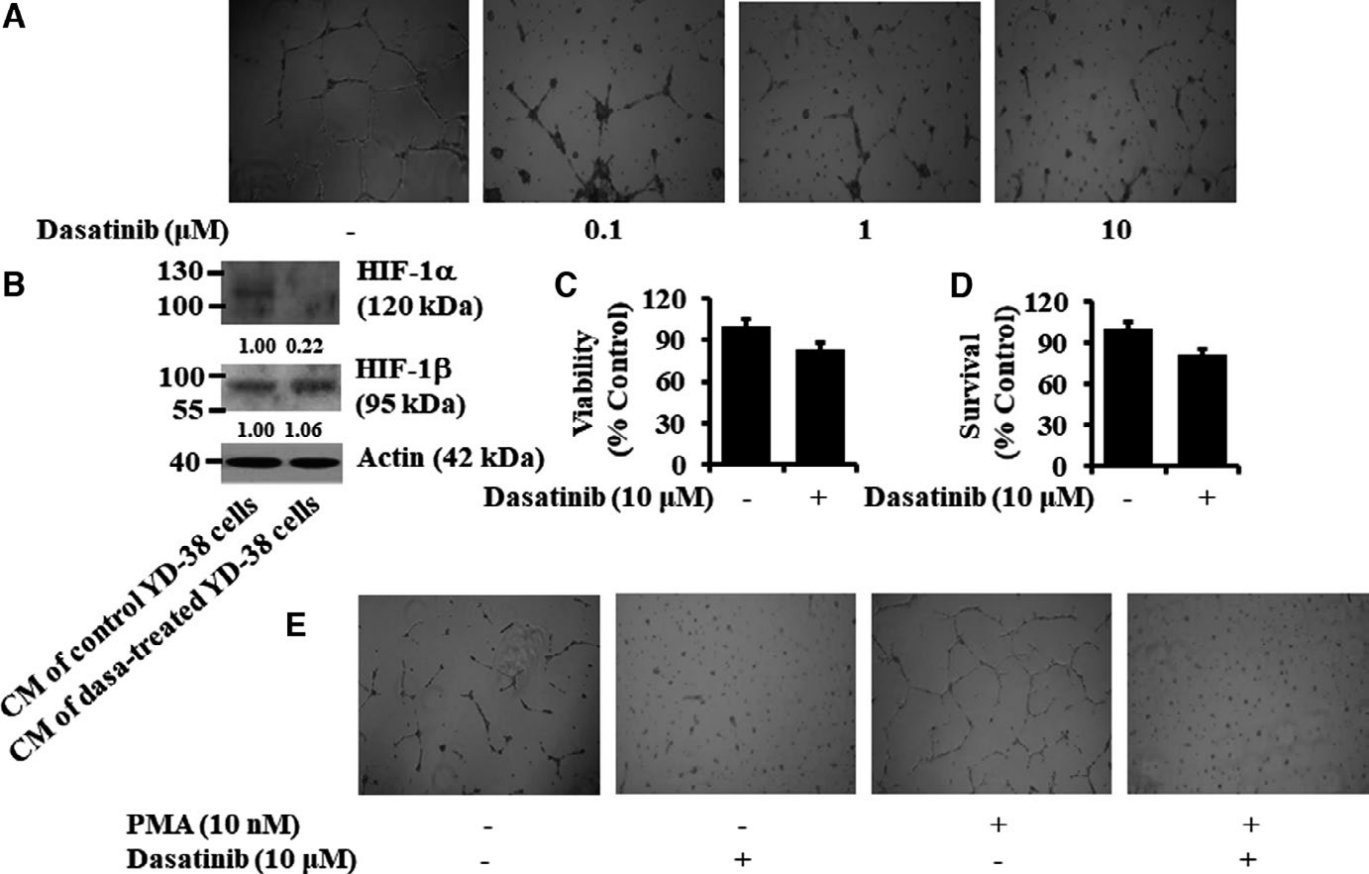
Effects of conditioned media (CM) from control‐ or dasatinib‐treated YD‐38 cells on tube formation, viability, survival, and HIF‐1α protein expression of HUVEC. (A‐B) YD‐38 cells were treated without or with dasatinib at the indicated doses for 24 h. The conditioned media (CM) was then collected and applied to HUVEC cultured in Matrigel‐coated plates for an additional 16 h. Changes in cell morphology (HUVEC tube formation) were captured using an inverted microscope (A). Whole‐cell lysates were prepared and analysed by Western blotting (B). (C‐D) HUVECs were treated without or with dasatinib (10 μM) for 16 h, followed by measurement of cell viability and cell survival by MTS (C) and cell count (D) assays, respectively. Data are the means ±SE of three independent experiments. (E) YD‐38 cells were treated without or with PMA (10 nM), a known inducer of HUVEC tube formation, in the absence or presence of dasatinib (10 µM) for 8 h. The conditioned media (CM) was then collected and applied to HUVEC cultured in Matrigel‐coated plates for an additional 16 h. Changes in cell morphology (HUVEC tube formation) were captured using an inverted microscope

We next investigated the effect of dasatinib on angiogenesis in vitro by measuring the tube formation of HUVEC exposed to the conditioned medium (CM) of YD‐38 cells treated without or with dasatinib. The tube formation of HUVEC was reduced in a dasatinib concentration‐dependent manner when treated with CM from dasatinib‐treated YD‐38 cells compared with control CM (Figure 8A). Moreover, there were substantial levels of HIF‐1α protein in HUVEC cultured with the CM from control YD‐38 cells, but there was no HIF‐1α protein in HUVEC cultured with the CM from dasatinib (10 µM)‐treated YD‐38 cells (Figure 8B). Expression levels of HIF‐1β and actin remained unchanged under these experimental conditions. We next determined whether dasatinib‐mediated inhibition of the tube formation in HUVEC is due to the drug's cytotoxicity. There was no significant change in the viability and survival of HUVEC treated for 24 hours without or with dasatinib at 10 µM, ruling out the possibility of the drug's cytotoxicity (Figure 8C,D). PMA is a known inducer of HUVEC tube formation.^42^ Treatment with PMA at 10 nM induced tube formation of HUVEC, which was also greatly attenuated by dasatinib treatment (Figure 8E).

### 3.9 Dasatinib also has anti‐proliferative, anti‐survival and pro‐apoptotic effects on tumorigenic HSC‐3 cells

**FIGURE 9:**
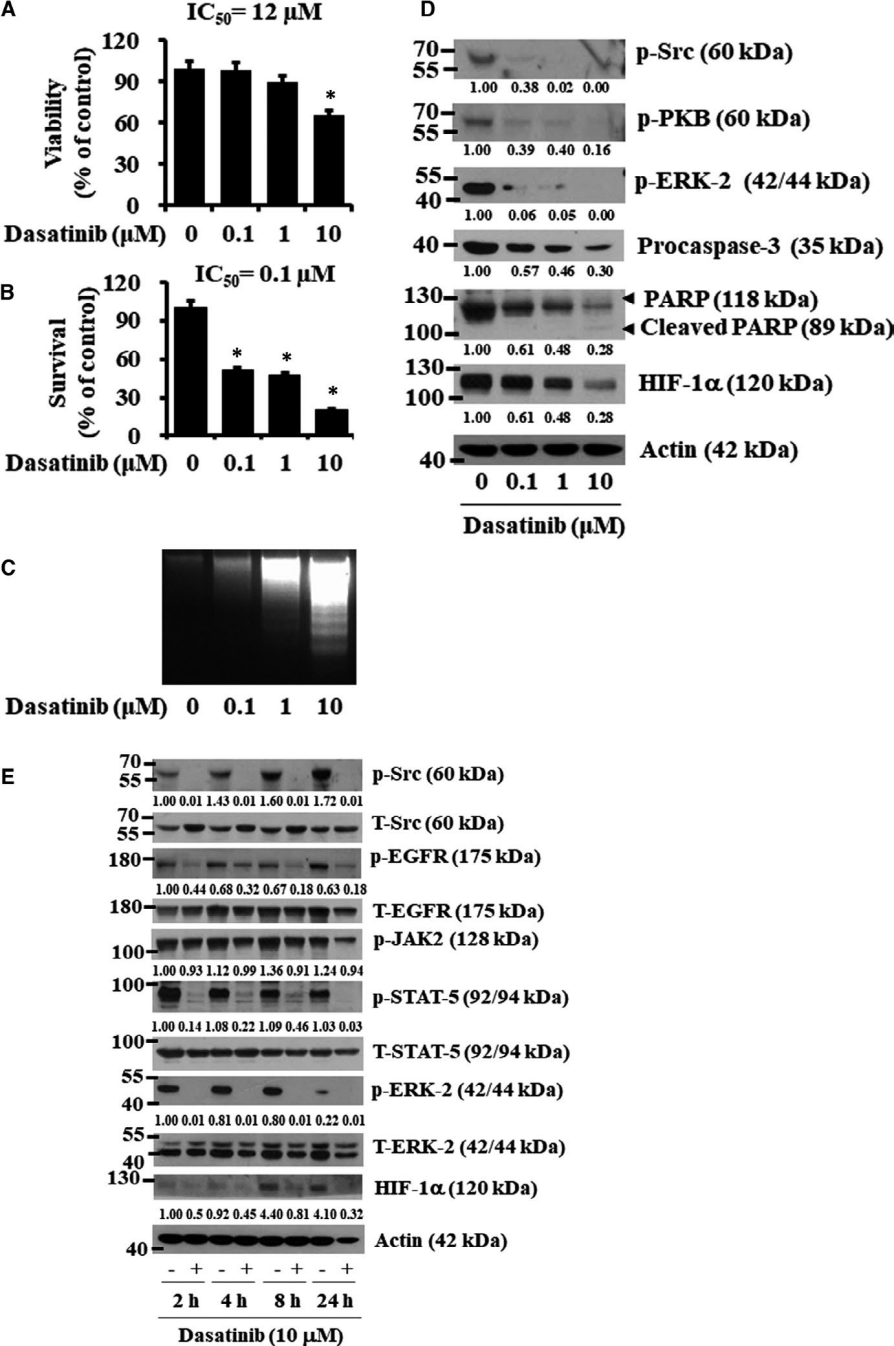
Effects of dasatinib on viability, survival, apoptosis, and expression and phosphorylation of cellular proteins in HSC‐3 cells. (A‐B) HSC‐3 cells were treated for 24 h with the indicated concentration of dasatinib, followed by measurement of cell viability and cell survival by MTS (A) and cell count (B) assays, respectively. Data are mean ±SE of three independent experiments. *P < .05 compared with the values of control (no drug). (C) HSC‐3 cells were treated with dasatinib at the indicated doses for 8 h. Extra‐nuclear fragmented DNA was extracted, and analysed on a 1.7% agarose gel. (D‐E) HSC‐3 cells were treated with dasatinib at the designated doses for 24 h (D) or at the indicated times (E). At the designated time point, whole‐cell lysates were prepared and analysed by Western blotting. Each picture is representative of three independent experiments

To see if dasatinib's anti‐proliferative, anti‐survival and pro‐apoptotic effects are limited to non‐tumorigenic YD‐38 cells, the effect of dasatinib on proliferation, survival and apoptosis of tumorigenic HSC‐3 cells was further investigated. Like YD‐38 cells, dasatinib treatment for 24 hours led to a concentration‐dependent reduction of HSC‐3 cell proliferation (Figure 9A) and survival (Figure 9B). Dasatinib treatment for 8 hours also led to a concentration‐dependent progressive accumulation of fragmented DNA in HSC‐3 cells (Figure 9C). Moreover, dasatinib treatment for 24 hours resulted in a dose‐dependent reduction of the phosphorylation levels of Src, PKB and ERK‐1/2 (Figure 9D). In addition, there was a concentration‐dependent decrease in levels of procaspase‐3, PARP and HIF‐1α in HSC‐3 cells treated with dasatinib for 8 hours. Results of time‐course experiments further revealed the ability of dasatinib (10 μM) to greatly reduce the phosphorylation levels of Src, EGFR, JAK2, STAT‐5, ERK‐1/2 and HIF‐1α in HSC‐3 cells for the time points tested (Figure 9E). Expression of control actin or total Src, EGFR, STAT‐5 and ERK‐2 proteins was not largely changed under these experimental conditions.

## 4 DISCUSSION

The anti‐cancer effect and the mode of action of dasatinib in human oral cancer are poorly understood. Here, we report that dasatinib has anti‐growth, anti‐angiogenic and pro‐apoptotic effects on both YD‐38 (non‐tumorigenic) and HSC‐3 (tumorigenic) human oral cancer cells, and these effects are mediated through control of the expression and phosphorylation of multiple targets including Src, EGFR, STAT‐3, STAT‐5, PKB, ERK‐1/2, S6, eIF‐2α, GRP78, caspase‐9/3, Mcl‐1 and HIF‐1α.

Dasatinib exerts anti‐tumour effects on blood malignancies and solid cancers by inhibiting cell proliferation/survival and inducing cell apoptosis.^19^, ^20^, ^21^, ^22^, ^43^, ^44^ It had been previously shown that dasatinib at 10 µM inhibits the viability and induces the apoptosis of HSC‐3 cells.^45^ Extending on this, we demonstrated that dasatinib at 10 µM markedly inhibits the proliferation and survival of YD‐8, YD‐10B, HSC‐3 and YD‐38 cells; the most sensitive being YD‐38 cells. Dasatinib's growth‐suppressive and apoptosis‐inducing effects are therefore not limited to HSC‐3 cells.

Dasatinib is an inhibitor of the non‐receptor tyrosine kinase Src.^19^, ^20^, ^46^ Src is highly expressed and phosphorylated (activated) in many solid tumours.^11^, ^12^, ^13^ High levels of phosphorylated Src are detected in most tongue cancer biopsies of human tongue cancer patients, and the outcome in patients with tongue cancer inversely correlates with Src hyperphosphorylation,^17^, ^18^ highlighting the prognostic role of Src overexpression/hyperactivation in tongue cancer. In this study, YD‐38 (and HSC‐3) cells express high levels of phosphorylated Src, but dasatinib at 10 µM almost completely down‐regulates them in these cells, supporting the drug's efficacy. Given that Src overexpression/hyperactivation contributes to cancer cell proliferation and survival,^14^, ^15^, ^16^ we further sought to explore the role of Src hyperphosphorylation in YD‐38 cells. Pharmacological inhibition of Src by PP1 (a selective inhibitor of Src) causes a substantial decrease in YD‐38 cell survival. These results confirm that Src is a survival factor in YD‐38 cells, and thus, dasatinib's anti‐growth effect on YD‐38 (and HSC‐3) is in part mediated through Src inhibition.

In agreement with previous studies,^45^ we found herein that dasatinib at 10 µM largely inhibits the phosphorylation of EGFR, PKB and ERK‐1/2 in both YD‐38 and HSC‐3 cells, further advocating dasatinib's multi‐targeted tyrosine kinase inhibition in both non‐tumorigenic and tumorigenic human oral cancer cells. Moreover, we demonstrated that respective pharmacological inhibition of EGFR, PI3K/PKB and ERK‐1/2 causes a substantial reduction of YD‐38 cell viability and survival. These results collectively suggest that EGFR, PI3K/PKB and ERK‐1/2 also act as survival factors in YD‐38 cells, and thus, dasatinib's anti‐growth effect on YD‐38 (and HSC‐3) cells is associated with inhibition of EGFR, PI3K/PKB and ERK‐1/2 signalling pathways.

STAT proteins including STAT‐3 and STAT‐5 are transcription factors involved in cancer cell proliferation and survival by regulating the transcription of cell cycle–related, survival‐related and apoptosis‐related genes.^47^ Notably, dasatinib inhibits STAT‐5 signalling associated with apoptosis in K562 CML cells,^48^ and it inhibits Src and STAT‐5 phosphorylation along with apoptosis induction in acute lymphoblastic leukaemia cells.^49^ These findings address that dasatinib‐mediated Src and STAT‐5 inhibition is crucial for the induction of apoptosis in these leukaemia cells. These findings address that dasatinib‐mediated Src and STAT‐5 inhibition is crucial for the induction of apoptosis in these leukaemia cells. The ability of dasatinib to regulate STAT proteins in human oral cancer cells is not known. Of interest, in the current study, dasatinib rapidly inhibits the phosphorylation of STAT‐3 and STAT‐5 without influencing their total protein expression in YD‐38 cells. Given that pharmacological inhibition of STAT‐3 and STAT‐5 by AG490 (a JAK/STAT inhibitor) results in a substantial reduction of YD‐38 cell survival, it is likely that STAT‐3 and STAT‐5 also facilitate the survival of YD‐38 cells, and thus, inhibition of STAT‐3 and STAT‐5 further contributes to dasatinib's growth‐suppressive and/or pro‐apoptotic effects on YD‐38 cells.

Src participates in much of the crosstalk between the receptor tyrosine kinases (RTKs) and cytoplasmic protein tyrosine kinases. Truly, a wealth of information illustrates that Src regulates EGFR, PKB and ERK‐1/2 in multiple cancers.^50^, ^51^, ^52^, ^53^ At present, the crosstalk between Src and the RTKs or cytoplasmic protein tyrosine kinases in human oral cancer cells remains elusive. Profoundly, we observed that knockdown of Src causes strong and selective inhibition of the phosphorylation of EGFR, STAT‐5, PKB and ERK‐1/2 in YD‐38 cells, but it does not induce cell apoptosis (data not shown). These results suggest that Src lies upstream of EGFR, STAT‐5, PKB and ERK‐1/2 in YD‐38 cells, and thus, dasatinib's anti‐growth effect on these cells is likely to be further mediated through Src inhibition–mediated suppression of EGFR, STAT‐5, PKB and ERK‐1/2.

The induction of cell apoptosis is mostly mediated via the intrinsic (mitochondrial)‐ and extrinsic (DR)‐mediated pathways.^54^ It is documented that members of the Bcl‐2 family, including Bcl‐2 and Mcl‐1, regulate apoptosis and caspase activation by regulating the mitochondrial membrane integrity,^55^ and the decline of the expression levels of Bcl‐2 members contributes to apoptosis induced by some anti‐cancer agents.^56^, ^57^ Human IAPs, including XIAP, additionally regulate apoptosis by inhibiting caspase‐9/3.^31^, ^58^, ^59^ Here, dasatinib induced the apoptosis of YD‐38 cells via the intrinsic apoptotic pathways but not an extrinsic pathway, as evidenced by nuclear DNA fragmentation, PARP cleavage and activation of caspase‐9 without affecting the expression of DR‐5, Bcl‐2 and XIAP and reducing the expression of Mcl‐1. Given that pharmacological blockade of caspase activation by z‐VAD‐fmk (a pan‐caspase inhibitor) greatly abolished the ability of dasatinib to trigger the apoptosis of YD‐38 cells, it is evident that the intrinsic activation of the caspase pathway is important for the induction of apoptosis in these cells. It is also likely that this Mcl‐1 down‐regulation may further contribute to caspase activation and induction of apoptosis in YD‐38 cells in response to dasatinib.

Induction of ER stress is common to many anti‐cancer drugs and/or agents.^60^, ^61^ ER stress occurs when cells have dysfunction of protein synthesis and excessive accumulation of misfolded proteins in the ER^62^ and high phosphorylation levels of eIF‐2α, a translation regulatory protein.^63^ The phosphorylation of eIF‐2α is indicative of its inactivation and global translational inhibition.^64^ It is known that the phosphorylation of S6, a ribosomal protein involved in translation, is closely associated with global translational activation.^35^ It is worth mentioning previous reports that the regulation of ER stress is identified as a mechanism for the anti‐cancer effects of dasatinib in head and neck cancers.^65^, ^66^ Since dasatinib increased the expression of GRP78 and the phosphorylation of eIF‐2α while decreasing the phosphorylation of S6 in YD‐38 cells, induction of ER stress and global translational inhibition in YD‐38 cells may therefore contribute to the drug's growth‐suppressive and pro‐apoptotic effects.

The transcription factor HIF‐1α is involved in tumour angiogenesis by regulating the transcriptional induction of angiogenesis‐related genes, such as VEGF, that contain HRE cis‐acting elements within their promoters.^34^, ^67^HIF‐1α is currently regarded as a prime target for anti‐cancer therapies.^68^, ^69^ At present, the mechanisms underlying high HIF‐1α protein expression and dasatinib regulation of HIF‐1α expression in oral cancer cells are poorly understood. In this study, we found high HIF‐1α protein expression in all four human oral cancer cell lines. Distinctly, the present study demonstrated that dasatinib greatly down‐regulated HIF‐1α protein in YD‐8, YD‐38 and HSC‐3, but not YD‐10B cells. These results indicate that dasatinib differentially regulates HIF‐1α expression in a cell type–dependent manner. A further striking finding herein is that dasatinib down‐regulates HIF‐1α at the protein, but not mRNA level, which is due to an increase in HIF‐1α protein turnover/destabilization through inhibition of Src, PI3K/PKB and ERK‐1/2. It is thus likely that dasatinib‐induced HIF‐1α protein down‐regulation and turnover in YD‐38 cells are associated with the drug's ability to inhibit these multi‐kinases.

In summary, we demonstrate that dasatinib has strong anti‐growth, anti‐angiogenic and pro‐apoptotic effects on YD‐38 and HSC‐3 cells, and these effects are mediated through the regulation of multiple cellular targets and pathways. This work suggests that dasatinib could be a possible regimen for the treatment of human oral cancer.

## CONFLICT OF INTEREST

The authors declare no conflict of interest.

## AUTHOR CONTRIBUTIONS

**Nam‐Sook Park:** Investigation (equal); Methodology (equal). **Yu‐Kyoung Park:** Investigation (equal); Methodology (equal). **Anil Kumar Yadav:** Data curation (equal); Investigation (equal); Writing‐original draft (equal). **Young‐Min Shin:** Formal analysis (equal); Validation (equal). **David Bishop Bailey:** Formal analysis (equal); Validation (equal); Writing‐review & editing (equal). **Jong‐Soon Choi:** Formal analysis (equal); Validation (equal). **Jong Wook Park:** Formal analysis (equal); Validation (equal). **Byeong‐Churl Jang:** Conceptualization (equal); Formal analysis (equal); Supervision (equal); Validation (equal); Visualization (equal); Writing‐original draft (equal).

## Supporting information

Table S1‐S2.

## Data Availability

The data that support the findings of this study are available from the corresponding author upon reasonable request.
