# Supplementary material for: Anti‐growth and pro‐apoptotic effects of dasatinib on human oral cancer cells through multi‐targeted mechanisms
Source: J Cell Mol Med. 2021 Jul 28;25(17):8300–11. doi: 10.1111/jcmm.16782 (PMC8419177; doi:10.1111/jcmm.16782)
Supplement: Supplementary file 1 — Table S1‐S2. [file JCMM-25-8300-s001.doc]

**Table S1: List of antibodies used for Western blot analysis.**

| **Antibodies** | **Dilution used** | **Source** | **Catalog no.** | |
| --- | --- | --- | --- | --- |
| ***Primary antibodies*** |  |  | |  |
| Procaspase-9 | 1:2,000 | Enzo Life Sciences | | ADI-AAM-139 |
| Procaspase-3 | 1:2,000 | Enzo Life Sciences | | ADI-AAP-113 |
| PARP | 1:2,000 | Roche Diagnostics | | 11835238001 |
| DR-5 | 1:2,000 | Novus Biologicals | | NBP1-45951 |
| p-Src (T416) | 1:2,000 | Cell signalling | | #2101 |
| Src | 1:2,000 | Cell signalling | | #2108 |
| p-EGFR (Y1068) | 1:2,000 | Cell signalling | | #2234 |
| EGFR | 1:2,000 | Cell signalling | | #2645 |
| p-PKB (S473) | 1:2,000 | Cell signalling | | #9271 |
| PKB | 1:2,000 | Cell signalling | | #9272 |
| p-ERK-1/2 (T202/Y204) | 1:2,000 | Cell signalling | | #9101 |
| ERK-1/2 | 1:2,000 | Cell signalling | | #9102 |
| p-eIF-2α (S51) | 1:2,000 | Abcam | | Ab32157 |
| eIF-2α | 1:2,000 | Cell signalling | | #9722 |
| p-S6 (S235/236) | 1:4,000 | Cell signalling | | #2211 |
| S6 | 1:4,000 | Cell signalling | | #2317 |
| p-STAT-5 (Y694) | 1:2,000 | Santa Cruz Biotechnology | | sc-101806 |
| STAT-5 | 1:2,000 | Santa Cruz Biotechnology | | sc-835 |
| Mcl-1 | 1:2,000 | Santa Cruz Biotechnology | | sc-819 |
| Bcl-2 | 1:2,000 | Santa Cruz Biotechnology | | sc-509 |
| p-JAK-2 (Y1007/1008) | 1:2,000 | Santa Cruz Biotechnology | | sc-21870 |
| p-STAT-3 (Y705) | 1:2,000 | Santa Cruz Biotechnology | | sc-8059 |
| STAT-3 | 1:2,000 | Santa Cruz Biotechnology | | sc-8019 |
| GRP-78 | 1:2,000 | Santa Cruz Biotechnology | | sc-13539 |
| HIF-1β | 1:2,000 | Santa Cruz Biotechnology | | sc-17811 |
| XIAP | 1:2,000 | R&D Systems | | AF8221 |
| HIF-1α | 1:2,000 | BD Bioscience | | 610958 |
| β-Actin | 1:10,000 | Sigma | | A5441 |
|  |  |  | |  |
|  |  |  | |  |
| ***Secondary antibodies*** |  |  | |  |
| Goat anti-rabbit IgG-HRP  Goat anti-mouse-IgG-HRP | 1:5,000  1:5,000 | Jackson ImmunoResearch  Laboratories  Jackson ImmunoResearch  Laboratories | | 111-035-045  115-035-062 |

**Table S2: Sequences of primers used for RT-PCR.**

| **Gene** | **Forward** | **Reverse** | |
| --- | --- | --- | --- |
| Mcl-1 | ATCTCTCGGTACCTTCGGGAG | ACCAGCTCCTACTCCAGCAAC |  |
| HIF-1α | CTCAAAGTCGGACAGCCTCA | CCCTGCAGTAGGTTTCTGCT |  |
| HIF-1β | GTGCGCACACATGCTTCTGT | CTTTATGGCCAAGTCTCGGGT |  |
| Actin | TCAAGATCATTGCTCCTCCTG | CTGCTTGCTGATCCACATCTG |  |
